# Saccharibacteria harness light energy using type-1 rhodopsins that may rely on retinal sourced from microbial hosts

**DOI:** 10.1038/s41396-022-01231-w

**Published:** 2022-04-19

**Authors:** Alexander L. Jaffe, Masae Konno, Yuma Kawasaki, Chihiro Kataoka, Oded Béjà, Hideki Kandori, Keiichi Inoue, Jillian F. Banfield

**Affiliations:** 1 Department of Plant and Microbial Biology, University of California, Berkeley, CA USA; 2 The Institute for Solid State Physics, The University of Tokyo, Kashiwa, Chiba, Japan; 3 PRESTO, Japan Science and Technology Agency, Kawaguchi, Saitama, Japan; 4 Department of Life Science and Applied Chemistry, Nagoya Institute of Technology, Showa-ku, Nagoya, Japan; 5 Faculty of Biology, Technion - Israel Institute of Technology, Haifa, Israel; 6 OptoBioTechnology Research Center, Nagoya Institute of Technology, Showa-ku, Nagoya, Japan; 7 Innovative Genomics Institute, University of California, Berkeley, CA USA; 8 Department of Earth and Planetary Science, University of California, Berkeley, CA USA; 9 Department of Environmental Science, Policy, and Management, University of California, Berkeley, CA USA

**Keywords:** Water microbiology, Microbial ecology

## Abstract

Microbial rhodopsins are a family of photoreceptive membrane proteins with a wide distribution across the Tree of Life. Within the candidate phyla radiation (CPR), a diverse group of putatively episymbiotic bacteria, the genetic potential to produce rhodopsins appears to be confined to a small clade of organisms from sunlit environments. Here, we characterize the metabolic context and biophysical features of Saccharibacteria Type-1 rhodopsin sequences derived from metagenomic surveys and show that these proteins function as outward proton pumps. This provides one of the only known mechanisms by which CPR can generate a proton gradient for ATP synthesis. These Saccharibacteria do not encode the genetic machinery to produce all-*trans*-retinal, the chromophore essential for rhodopsin function, but their rhodopsins are able to rapidly uptake this cofactor when provided in experimental assays. We found consistent evidence for the capacity to produce retinal from β-carotene in microorganisms co-occurring with Saccharibacteria, and this genetic potential was dominated by members of the *Actinobacteria*, which are known hosts of Saccharibacteria in other habitats. If Actinobacteria serve as hosts for Saccharibacteria in freshwater environments, exchange of retinal for use by rhodopsin may be a feature of their associations.

## Introduction

The sun is the dominant source of energy on Earth, and many organisms have evolved ways to use light. Only recently was it suggested that some members of the candidate phyla radiation (CPR)—a highly diverse group of bacteria originally detected by 16 S rRNA sequencing [1] and subsequently characterized by genome-resolved metagenomics [2, 3]—may be able to use rhodopsins for proton translocation and thus energy generation [4, 5]. However, experimental evidence supporting this function was lacking. Here, we biophysically characterize rhodopsins from putatively symbiotic Saccharibacteria (TM7 lineage of CPR) and explore their relevance for metabolism. We also consider how rhodopsins may play a role in the interactions between Saccharibacteria and their putative microbial hosts in sunlit environmental microbiomes.

## Results

**Fig. 1:**
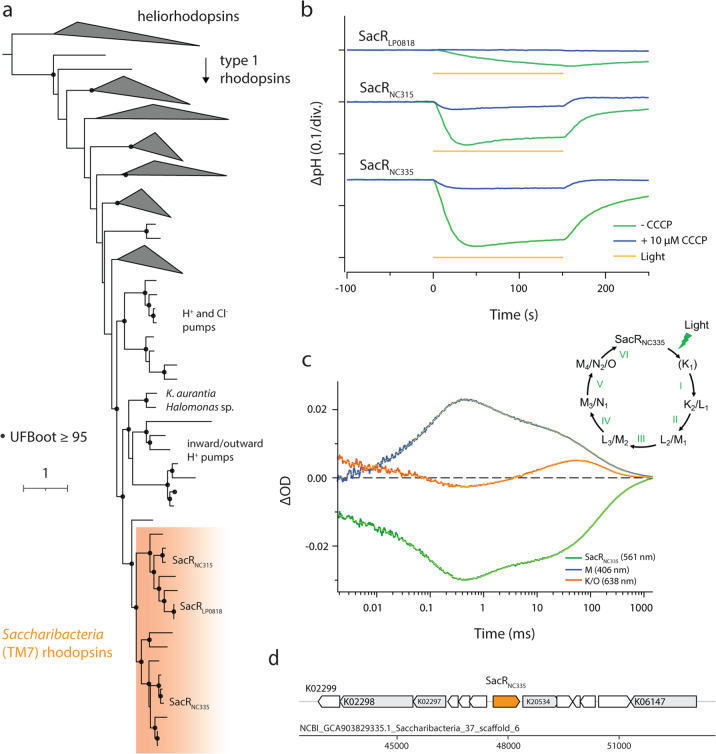
Characteristics of Saccharibacteria rhodopsins (SacRs). **a** Rhodopsin protein tree indicating that SacRs from freshwater lakes form a broad clade of proton pumps. **b** The ion-pumping activity of SacRs. Blue and green lines indicate the pH change with and without 10 μM CCCP, respectively. Yellow bars indicate the period of light illumination. **c** Time evolution of transient absorption changes of SacR_NC335_ in 100 mM NaCl, 20 mM HEPES–NaOH, pH 7.0, and POPE/POPG (molar ratio 3:1) vesicles with a lipid to protein molar ratio = 50. Time evolution at 406 nm (blue, representing the M accumulation), 561 nm (green, representing the bleaching of the initial state and the L accumulation), and 638 nm (red, representing the K and O accumulations). Yellow lines indicate fitting curves by a multi-exponential function. Inset: The photocycle of SacR_NC335_ based on the fitting in (**c**) and a kinetic model assuming a sequential photocycle. The lifetime (*τ*) of each intermediate is indicated by numbers as follow (mean ± S.D., fraction of the intermediate decayed with each lifetime in its double exponential decay is indicated in parentheses): I: *τ* = 1.7 ± 0.3 μs (42%), *τ* = 13 ± 1.8 μs (58%), II: *τ* = 118 ± 2 μs, III: *τ* = 1.6 ± 0.1 ms, IV: *τ* = 23.5 ± 1.0 ms, V: *τ* = 98.4 ± 6.4 ms (56%), *τ* = 384 ± 18 ms (44%). **d** Genomic context of SacR_NC335_. Neighboring genes with above-threshold KEGG annotations are indicated in gray with the highest-scoring HMM model. Genes without KEGG annotations are indicated in white.

Phylogenetic placement of Saccharibacteria rhodopsins (SacRs) shows that these sequences form a sibling clade to characterized light-driven inward and outward H^+^ pumps (Fig. 1a). We selected three phylogenetically diverse SacRs from freshwater lakes (Table S1) and two related, previously uncharacterized sequences from the *Gammaproteobacteria* (*Kushneria aurantia* and *Halomonas* sp.) for synthesis and functional characterization (highlighted in Fig. 1a). All sequences have Asp–Thr–Ser (DTS) residues at the positions of D85–T96–D96 of bacteriorhodopsin (BR) in the third transmembrane helix (Fig. S1). These residues are known as the triplet DTD motif and represent key residues for proton pumping function in BR [6].

Proton transport assays for the SacRs and Gammaproteobacteria proteins expressed in *Escherichia coli* showed marked decrease of external pH upon light illumination (Fig. 1b and Fig. S2), indicating that these proteins are light-driven outward H^+^ pumps. The pH decrease was almost eliminated after adding the protonophore carbonyl cyanide m-chlorophenyl hydrazone (CCCP), which dissipates the H^+^ gradient, confirming that it was indeed formed upon illumination (Fig. 1b and Fig. S2). We also characterized the absorption spectra and the photocycle of the SacRs, showing that the three rhodopsins have an absorption peak around 550 nm (Fig. S3). The photocycle of the SacRs, determined by measuring the transient absorption change after nanosecond laser pulse illumination (Fig. 1c and Fig. S4), displays a blue-shifted M intermediate that represents the deprotonated state of the retinal chromophore. This has been observed for other H^+^ pumping rhodopsins [7, 8] and indicates that the proton bound to retinal is translocated during pumping.

Given that SacRs function as outward proton pumps, we searched Saccharibacteria genomes for the F_1_F_o_ ATP synthase that would be required to harness the generated proton motive force for ATP synthesis. HMM searches showed that all genomes encoded the complete ATP synthase gene cluster and, furthermore, had c subunits with motifs consistent with H^+^ binding, instead of Na^+^ binding (Table S1 and Fig. S5). Together, our experimental and genomic analyses strongly suggest that some Saccharibacteria utilize rhodopsins for auxiliary energy generation in addition to their core fermentative capacities [6].

Retinal is the rhodopsin chromophore that enables function of the complex upon illumination [9]. We found no evidence for the presence of β-carotene 15,15’-dioxygenase (*blh*), which produces all-*trans*-retinal (ATR) from β-carotene, in Saccharibacteria genomes encoding rhodopsin. This absence was likely not due to genome incompleteness, as genomic bins were generally of high quality (79–98% completeness, Table S1) and rhodopsin genomic loci were well-sampled. Additionally, no conserved hypothetical proteins were present in these regions, where *blh* is often found [10] (Fig. 1d, Fig. S6 and Table S2). As SacRs do contain the conserved lysine for retinal binding [4], we instead hypothesized that Saccharibacteria may uptake retinal from the environment, as has been previously observed for other microorganisms encoding rhodopsin but also lacking *blh* [11, 12].

**Fig. 2:**
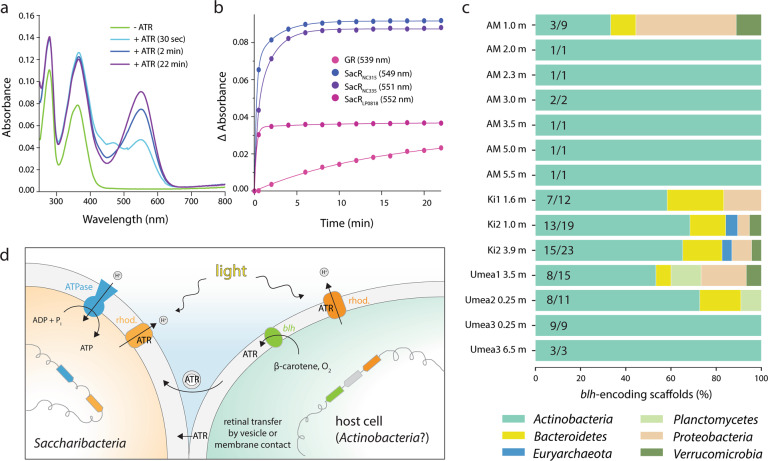
Binding of retinal by Saccharibacteria rhodopsins and context for biosynthesis. **a** UV-visible absorption spectra showing the regeneration of retinal binding to SacR_NC335_ and GR in 20 mM HEPES–NaOH, pH 7.0, 100 mM NaCl and 0.05% n-dodecyl-β-D-maltoside (DDM). In SacR_NC335_, a peak around 470 nm was transiently observed in the spectrum 30 s after the addition of ATR, suggesting that an intermediate species appears during the retinal incorporation process that involves formation of the Schiff base linkage. **b** Time evolution of visible absorption representing retinal binding to apo-protein. Numbers in parentheses in the legend indicate the absorption maxima of each rhodopsin. **c** Genetic potential for β-carotene 15,15’-dioxygenase (*blh*) production in freshwater lake metagenomes where SacRs are found. Fractions indicate the number of *blh*-encoding scaffolds taxonomically affiliated with the *Actinobacteria* in each sample. **d** Conceptual diagram illustrating potential retinal exchange between Saccharibacteria and host cells. ATR all-*trans-*retinal, GR *Gloeobacter* rhodopsin, AM Alinen Mustajärvi, Ki Kiruna, rhod. rhodopsin.

We tested the ability of SacR proteins to bind ATR from an external source by performing a retinal reconstitution assay. In contrast to the proton transport assays, where rhodopsin was expressed in the presence of ATR, here ATR was dissociated from the purified complex and the visible absorbance of rhodopsin was measured upon re-addition of ATR [13]. Both *Gloeobacter* rhodopsin (GR), a typical Type-1 outward H^+^ pump, and SacRs showed an increase in absorption in the visible region with time after the addition of ATR (Fig. 2a and Fig. S7). For all SacRs, the binding of ATR by their apoprotein was saturated within 30 sec after retinal addition (Fig. 2b), indicating that SacR is able to be efficiently functionalized using externally derived ATR. The observed reconstitution rate is substantially faster than that of GR ( > 20 min) and comparable to that of heliorhodopsin, which is used by other microorganisms also lacking a retinal synthetic pathway and rapidly binds ATR through a small opening in the apoprotein [12]. In the structure of SacR_NC335_ modeled by Alphafold2 [14, 15], a similar hole is visible in the protein moiety constructing the retinal binding pocket (Fig. S8). Hence, SacRs may also bind retinal through this hole in a similar manner to *T*aHeR (heliorhodopsin).

Saccharibacteria with rhodopsin must obtain retinal from other organisms. To evaluate possible sources of ATR, we investigated the genetic potential for retinal biosynthesis in 15 subarctic and boreal lakes [16] where Saccharibacteria with rhodopsin were present (Fig. S9). *Blh*-encoding scaffolds were found in 14 of the 15 metagenomes profiled (~93%) and, in nearly all cases, these scaffolds derived from Actinobacteria (Fig. 2c and Table S3). This is intriguing because Actinobacteria are known to be hosts of Saccharibacteria in the human microbiome [17, 18] and potentially more generally [4, 19]. BLAST searches against genome bins from the same samples indicated that these Actinobacteria were members of the order *Nanopelagicales* (Table S3) and often encode a rhodopsin (phylogenetically distinct from SacRs) in close genomic proximity to *blh* genes (Table S4). HMM searches revealed that these genomes also harbor homologs of the *crtI*, *crtE*, *crtB*, and *crtY* genes necessary for β-carotene production [20].

## Discussion

Here, we add to growing evidence that DTS-motif rhodopsins can function as outward H^+^ pumps [21] and infer that Saccharibacteria use them to establish a proton gradient for energy generation, given a source of ATR and light. This is one of the very few known ways that any CPR organism can pump protons across the membrane. However, the source of ATR enabling the function of Saccharibacteria rhodopsins is unclear. While there is precedent for external supply of ATR to functional rhodopsins in other bacteria [11, 12], the mechanism by which this hydrophobic compound is transferred to the membrane of such bacteria is also unknown.

Experimental co-cultures of Saccharibacteria with Actinobacteria from multiple microbiome types [18, 19] suggest that a host bacterium for the Saccharibacteria studied here may be the source of ATR. We infer that these hosts are co-occurring *Nanopelagicales* Actinobacteria that dominate retinal production in microbial communities containing Saccharibacteria with rhodopsin. These *Nanopelagicales* bacteria are sufficiently abundant to represent plausible hosts (Fig. S10a) and have average genome sizes of approximately 1.25 Mbp (Fig. S10b). This is substantially smaller than known hosts of Saccharibacteria from other environments (Fig. S10b) but still larger than Saccharibacteria themselves (~0.78 Mbp, on average). However, the genetic requirements to host CPR symbionts is currently unknown.

If *Nanopelagicales* bacteria are indeed the hosts of freshwater Saccharibacteria with rhodopsin, then retinal produced by the former from β-carotene could be transferred to the latter either by membrane contact, a common feature in imaged CPR-host interactions [17, 22], or possibly via extracellular vesicles (Fig. 2d). ATR produced by Actinobacteria is required for their own rhodopsins [11] (Fig. 2d), but it is conceivable that they produce ATR in excess to deliberately supply Saccharibacteria symbionts, possibly to ensure interdependence. Alternatively, Saccharibacteria scavenge ATR. Regardless of the source organism and ATR transfer mechanism, our analyses suggest a new aspect of Saccharibacteria lifestyles, in which they employ rhodopsins and externally derived retinal to produce energy via phototrophy.

## Supplementary information

Supplemental Methods and Figures

Supplemental Tables

## Data Availability

All accession information for the genomes and metagenomic samples analyzed in this study are listed in the Supplementary Tables. Additional files (including the masked rhodopsin alignment and maximum likelihood tree), supplementary tables, and custom code for the described analyses are also available on Zenodo (10.5281/zenodo.6038621).
