# Supplementary material for: Saccharibacteria harness light energy using type-1 rhodopsins that may rely on retinal sourced from microbial hosts
Source: ISME J. 2022 Apr 19;16(8):2056–9. doi: 10.1038/s41396-022-01231-w (PMC9296517; doi:10.1038/s41396-022-01231-w)
Supplement: Supplementary file 1 — Supplemental Methods and Figures [file 41396_2022_1231_MOESM1_ESM.pdf]

Saccharibacteria harness light energy using Type-1 rhodopsins that may rely on retinal sourced from microbial hosts

Alexander L. Jaffe[1 †], Masae Konno[2,3 †], Yuma Kawasaki[2], Chihiro Kataoka[4], Oded Béjà[5], Hideki Kandori[4,6], Keiichi Inoue[2]*, Jillian F. Banfield[7,8,9]*

† These authors contributed equally to this work.
* Corresponding author emails: inoue@issp.u-tokyo.ac.jp, jbanfield@berkeley.edu

Supplementary Methods

Phylogenetic tree building

Rhodopsin protein sequences from Jaffe et al. 2021 [1] were combined with additional reference sequences from public databases as well as a set of newly-reported sequences from freshwater Saccharibacteria for phylogenetic analysis [2]. Also included was a representative rhodopsin sequence from a freshwater Saccharibacteria genome derived from a metagenomic survey of the meromictic Lac Pavin. Identical sequences were removed using usearch (*-cluster_fast -id 1*) [3] and aligned using ClustalW [4]. Alignment of the 1st, 2nd, and 5th transmembrane helices was corrected manually. Finally, a maximum likelihood tree was inferred using IQTree (*-m TEST -bb 1000*) [5]. The corrected alignment and maximum likelihood tree are available on Zenodo (see **Data and software availability** statement).

Sequence synthesis and expression

Three phylogenetically diverse Saccharibacteria rhodopsin sequences and two sequences from the *Gammaproteobacteria* were selected for experimental characterization. To ensure the accuracy of protein predictions, metagenomic reads for each sample were mapped back to assembled Saccharibacteria contigs with rhodopsin using bowtie2 (default parameters) [6]. Read mapping for each contig was examined manually using Geneious.

Genes encoding each rhodopsin were artificially synthesized with codon optimization for an *Escherichia coli* expression (Genscript, Piscataway, NJ, USA) and cloned into *Nde*I-*Xho*I site of pET21a(+) vector (Novagen, Merck KGaA, Germany). The constructed plasmids were transformed into *E. coli* C43(DE3) strain (Lucigen, WI, USA). The protein expression was induced by 0.1 mM isopropyl-β-D-thiogalactopyranoside (IPTG) in the presence of 10 μM ATR (Toronto Research Chemicals, Canada) for 4 h at 37 °C. The expressed proteins had a 6 × His-tag on the C-terminus.

Proton transport, absorption spectra, and photocycle of Saccharibacteria rhodopsins

Light-driven proton transport was measured according to previously reported protocol [7] . Briefly, the number of *E. coli* cells expressing each rhodopsin was estimated by their optical density of the culture at 660 nm (OD_{660}). In the following measurements, the equivalent of 15

OD_{660}•mL (OD_{660} × total volume (ml) of the cell suspension) cell amount was used. The cells were collected by centrifuge (4,800 × *g*, 2 min, 20 °C), washed once and equilibrated three times with unbuffered 100 mM NaCl solution. The equilibrated cells were resuspended by unbuffered 100 mM NaCl solution and adjusted at OD_{660} = 2. The cell suspension was placed in the dark at 20 °C and illuminated at λ > 500 nm using a 300 W xenon light source (MAX-303, Asahi Spectra, Japan) through a long pass filter (Y-52; AGC Techno Glass, Japan) and a heat-absorbing filter (HAF-50S-50H; SIGMAKOKI, Japan). The light-induced changes in pH were measured using a pH electrode (9618S-10D; HORIBA, Japan). Measurements were repeated under the same condition after the addition of 10 µM CCCP.

Proteins were purified using a 5 mL Co^{2+}-NTA column (HiTrap TALON crude; Cytiva,Tokyo, Japan) on an ÄKTA start protein purification system (Cytiva, MA). The rhodopsin-expressing cells were harvested and resuspended in a buffer containing 50 mM Tris–HCl (pH 8.0) and 5 mM $MgCl_2$. The harvested cells were disrupted by sonication (Ultrasonic Homogenizer VP-300N, TAITEC, Japan). The membrane fraction was collected by ultracentrifugation (CP80NX, Eppendorf Himac Technologies, Japan) at 142,000 × *g* for 1 h. The proteins were solubilized in a buffer containing 50 mM Tris–HCl (pH 7.5), 300 mM NaCl, 5 mM Imidazole and 3 % n-Dodecyl-β-D-maltopyranoside (DDM). Solubilized proteins were separated from insoluble fractions by ultracentrifugation at 142,000 × g for 1 h. After loading the solubilized proteins on Co^{2+}-NTA column, the column was washed with a buffer containing 50 mM Tris–HCl (pH 7.5), 300 mM NaCl, 15 mM Imidazole and 0.1 % DDM. The His-tagged proteins were eluted with a buffer containing 50 mM Tris–HCl (pH 7.5), 300 mM NaCl, 500 mM Imidazole and 0.1 % DDM. The eluted proteins were dialyzed using buffer 20 mM HEPES–NaOH (pH 7.0), 100 mM NaCl, 0.05 % DDM to remove imidazole. Absorption spectra were recorded with a UV–vis spectrometer (V-750, JASCO, Japan).

For the transient absorption measurement by laser flash photolysis method, purified SacRs were reconstituted into a mixture of 1-palmitoyl-2-oleoyl-phosphatidyl-ethanolamine (POPE, Avanti Polar Lipids, AL) and 1-palmitoyl-2-oleoyl-sn-glycero-3-phosphoglycerol (POPG, sodium salt, Avanti Polar Lipids, AL) (molar ratio = 3:1), with a protein to lipid molar ratio of 1:50, in buffer containing 20 mM HEPES–NaOH, 100 mM NaCl, pH 7.0. The sample was illuminated with a beam of the second harmonics of a nanosecond-pulsed Nd-YAG laser (λ = 532 nm, 1.4–0.5 Hz, INDI40, Spectra-Physics, CA) with a pulse energy of 4.5 mJ/cm^2/pulse. The transient absorption spectra were obtained by monitoring the intensity change of white-light from a Xe-arc lamp (L9289-01, Hamamatsu Photonics, Japan) passed through the sample, with an ICCD linear array detector (C8808-01, Hamamatsu Photonics, Japan). To increase the signal-to-noise (S/N) ratio, 10–30 identical spectra were averaged and a singular value decomposition analysis was applied. To measure the time-evolution of transient absorption change at specific wavelengths with better time resolution, the light of Xe-arc lamp (L9289-01, Hamamatsu Photonics, Japan) was monochromated by monochromators (S-10, SOMA OPTICS, Japan) and the change in the intensity after the photo-excitation was monitored with a photomultiplier tube (R10699, Hamamatsu Photonics, Japan) equipped with a notch filter (532 nm, bandwidth = 17 nm, Semrock, NY) to remove the scattered pump pulse. To increase S/N ratio, 50–100 signals were averaged.

Analysis of metabolism and genomic context of Saccharibacteria rhodopsins

Proteins were predicted for all rhodopsin-encoding Saccharibacteria bins using Prodigal [8] (single mode) and subjected to annotation with KofamScan [9]. HMM hits were initially filtered to those with an e-value ≤ $1x10^{-6}$ and the highest-scoring hit for each open reading frame was selected. These results were used to identify candidate gene clusters encoding the F_1F_o ATP synthase in each genome. The presence of all subunits was verified manually using a combination of KEGG annotations and additional BLAST searches. C subunit protein sequences of these gene clusters were aligned with reference sequences and manually examined for the Q…ES/T motif indicative of Na^+ binding [10]. Finally, genomic context of the three experimentally characterized Saccharibacteria rhodopsins was visualized using gggenes (https://github.com/wilkox/gggenes). KoFamScan annotations were secondarily filtered to those with e-value ≤ $1x10^{-20}$ and displayed for those open reading frames within 5 kilobases upstream or downstream of rhodopsin sequences.

Community metabolic potential for retinal biosynthesis

To examine the composition and metabolic potential of communities with rhodopsin-encoding Saccharibacteria, we used a subset of Saccharibacteria genomes reconstructed from a prior metagenomic survey of boreal/subarctic lakes [11]. We identified the sample (or samples, in the case of co-assemblies) from which each genome was constructed and mapped quality-filtered metagenomic reads from these samples back to the corresponding genome using bowtie2 (default parameters). We next used inStrain [12] to compute per-gene read coverage and breadth (fraction of gene covered) for each genome-sample pair. Samples where any Saccharibacteria rhodopsin gene attained 80% coverage breadth (or better) were retained for downstream analyses (Fig. S9).

First, quality-filtered metagenomic reads from each sample were assembled using MEGAHIT [13] (*--min-contig-len 1000*) and all proteins were predicted using Prodigal (meta mode). Predicted metagenomic proteins were searched for β-carotene 15,15'-dioxygenase (*blh,* K21817) using hmmsearch. Proteins with high-scoring hits to the K21817 HMM (at or above the model-specific score threshold) were subjected to secondary annotation with KoFamScan to verify that there were no higher-scoring HMM models.

Next, we determined taxonomic affiliation for metagenomic contigs with *blh* genes by comparing all predicted proteins on these contigs against UniRef100 using a custom DIAMOND database (diamond blastp) [14]. Hits were filtered to those with ≥70% coverage of the query sequence and an e-value ≤ $1x10^{-5}$. We retrieved taxonomic affiliation for above-threshold hits and computed the percentage of genes on each scaffold with highest similarity to various bacterial phyla. Two scaffolds with ambiguous taxonomic affiliation were investigated manually using information from UniRef and BLAST searches; all other scaffolds were assigned to the phylum-level lineage with the highest percentage of hits across annotated genes.

Finally, we attempted to match *blh*-encoding scaffolds from the *Actinobacteria* with binned genome sequences from the same set of metagenomic samples. Moderate to high-quality Actinobacteria MAGs from [11] were downloaded and used to construct a custom BLAST database against which *blh*-encoding scaffolds from above were compared. Local alignments were filtered to those with >90% identity and >50% coverage of the query scaffolds. *Blh*-encoding scaffolds with above-threshold genome alignments were assigned bin-level taxonomy reported by the original study [11]. Actinobacteria bins were then searched for *blh*, rhodopsin, and genes involved in β-carotene synthesis using KoFamScan annotations of predicted proteins as above, except employing an e-value threshold of $1x10^{-5}$ to capture more distant/shorter homologs of lycopene beta-cyclase (K22502).

Retinal reconstitution assays

SacRs and GR apo-proteins were prepared by incubating purified proteins with 500 mM hydroxylamine (HA) and then illuminating at λ > 500 nm from the output of a 300 W xenon light source (MAX-303, Asahi Spectra, Japan) through a long pass filter (Y-52; AGC Techno Glass, Japan) and a heat-absorbing filter (HAF-50S-50H; SIGMAKOKI, Japan) until the proteins were completely bleached. Absorption changes representing the bleaching of rhodopsins by hydroxylamine were monitored using a UV-vis spectrometer (V-750, JASCO, Japan). Apo-protein samples were washed five times by ultrafiltration (Amicon ultra 30,000 NMWL, Merck Millipore, Germany) with a buffer (20 mM HEPES-NaOH, pH 7.0, containing 100 mM NaCl and 0.05 % DDM) to remove retinal oxime and unreacted HA. To reconstitute the apo-proteins with ATR, the apo-proteins (~ 1.7 μM) were incubated with 1.5 molar equiv. of ATR at 25 °C in the dark. The absorption changes were monitored during the retinal reconstitution.

Structural modeling of SacRs

Structure of $SacR_{NC335}$ was modeled by AlphaFold2 [15] using an unmodified version of ColabFold [16], with the default MSA pipeline, a MMseqs2 [17] search of UniRef [18] and environmental sample sequence databases [19, 20].

Analysis of community composition and genome size

Community composition of the metagenome samples described above was analyzed using GraftM [21] and a previously published gene package built from diverse ribosomal protein S3 (rpS3) sequences [22]. Counts of rpS3 reads corresponding to Saccharibacteria, *Nanopelagicales* Actinobacteria, and other Actinobacteria were tallied and expressed as a fraction of all rps3 reads identified in each sample. Genome/bin sizes for Saccharibacteria and *Nanopelagicales* bacteria from this study were computed simply by summing the lengths of member contigs. Known hosts of Saccharibacteria from other environments were drawn from previous studies [1, 23] and genome sizes were computed in the same fashion.

Supplementary Figures

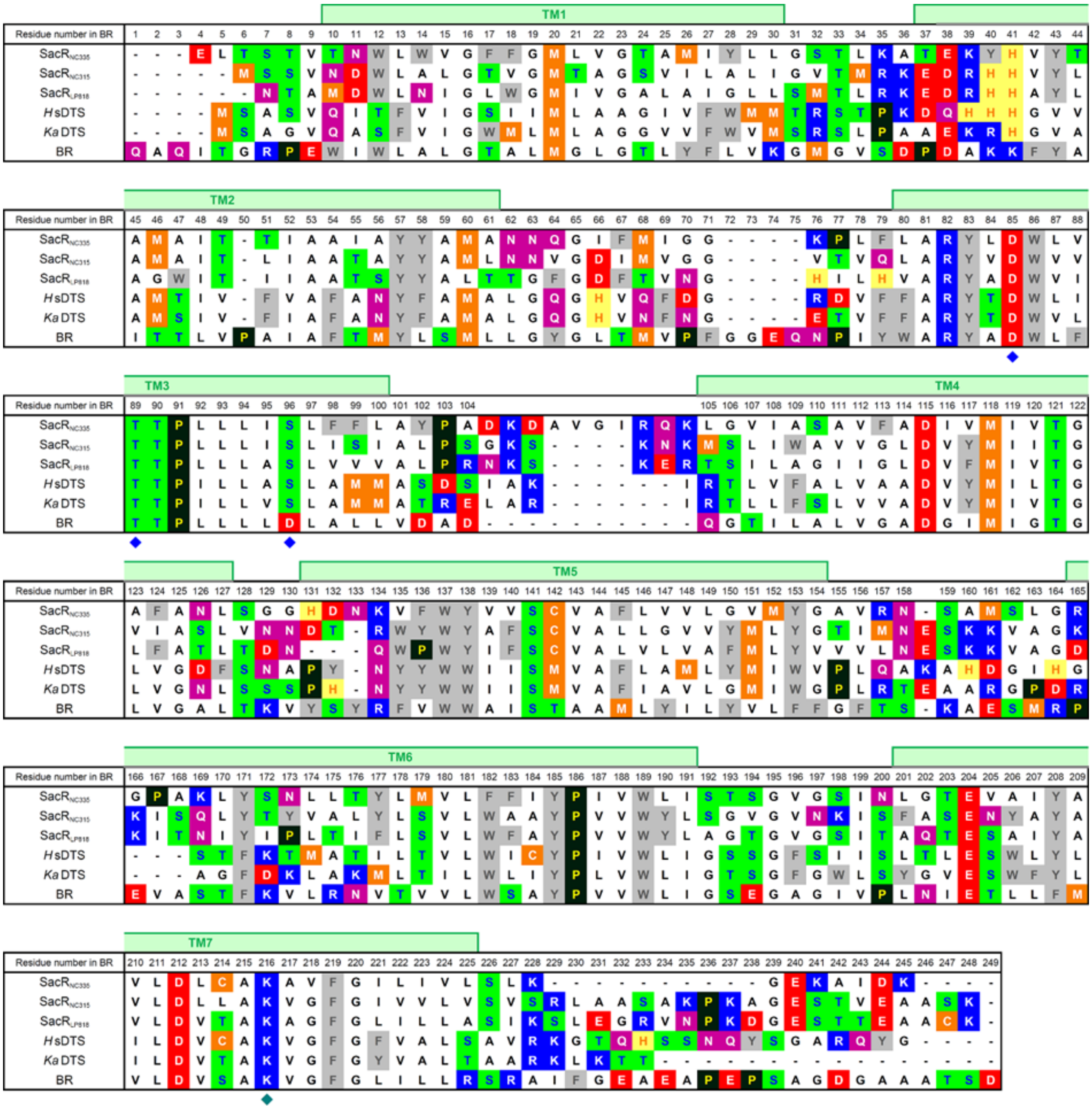

Figure S1. Amino acid sequence alignment of SacR with *Hs*DTS, *Ka*DTS, and BR. The positions of the seven transmembrane helices (TM1–TM7) in the X-ray crystallographic structure of BR (PDB ID: 1M0L) are indicated by green rectangles. The triplet motif in TM3 and retinal-binding lysine are indicated by blue and green diamonds, respectively.

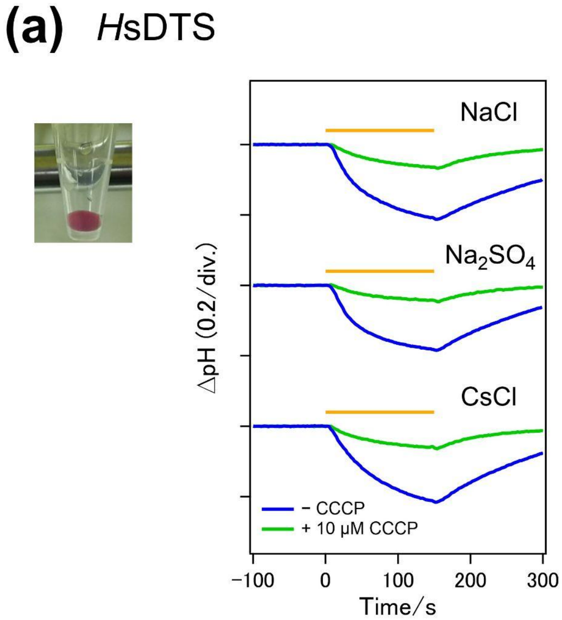

Figure S2. Proton transport assay of *Hs*DTS (*Halomonas* sp.) and *Ka*DTS (*Kusheria aurantia*). Light-induced pH change in external solvent of *E. coli* cells expressing *Hs*DTS (**a**) and *Ka*DTS (**b**) without (blue) and with (green) 10 µM CCCP. Light ($\lambda \geq 500$ nm) was illuminated for a period indicated by yellow bars. Pictures of *E. coli* cell pellets expressing rhodopsins are shown.

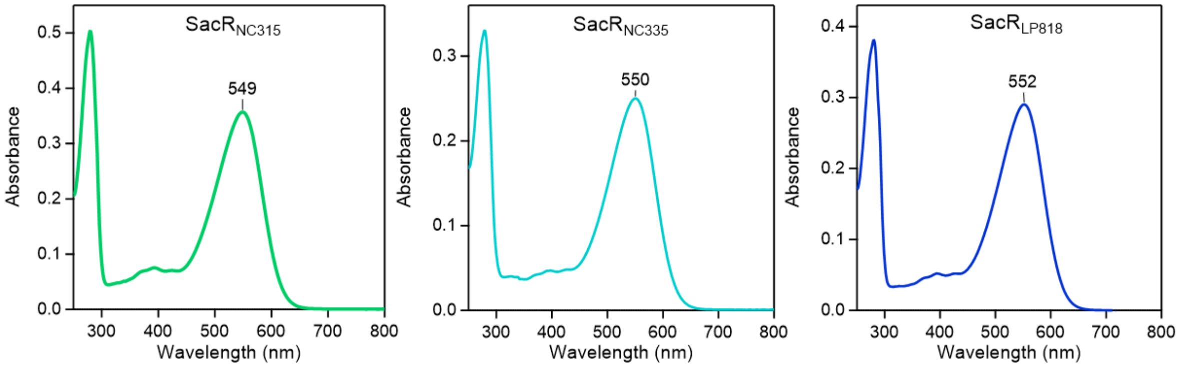

Figure S3. UV visible absorption spectra of SacRs in 100 mM NaCl, 20 mM HEPES–NaOH, pH 7.0, 0.05% DDM.

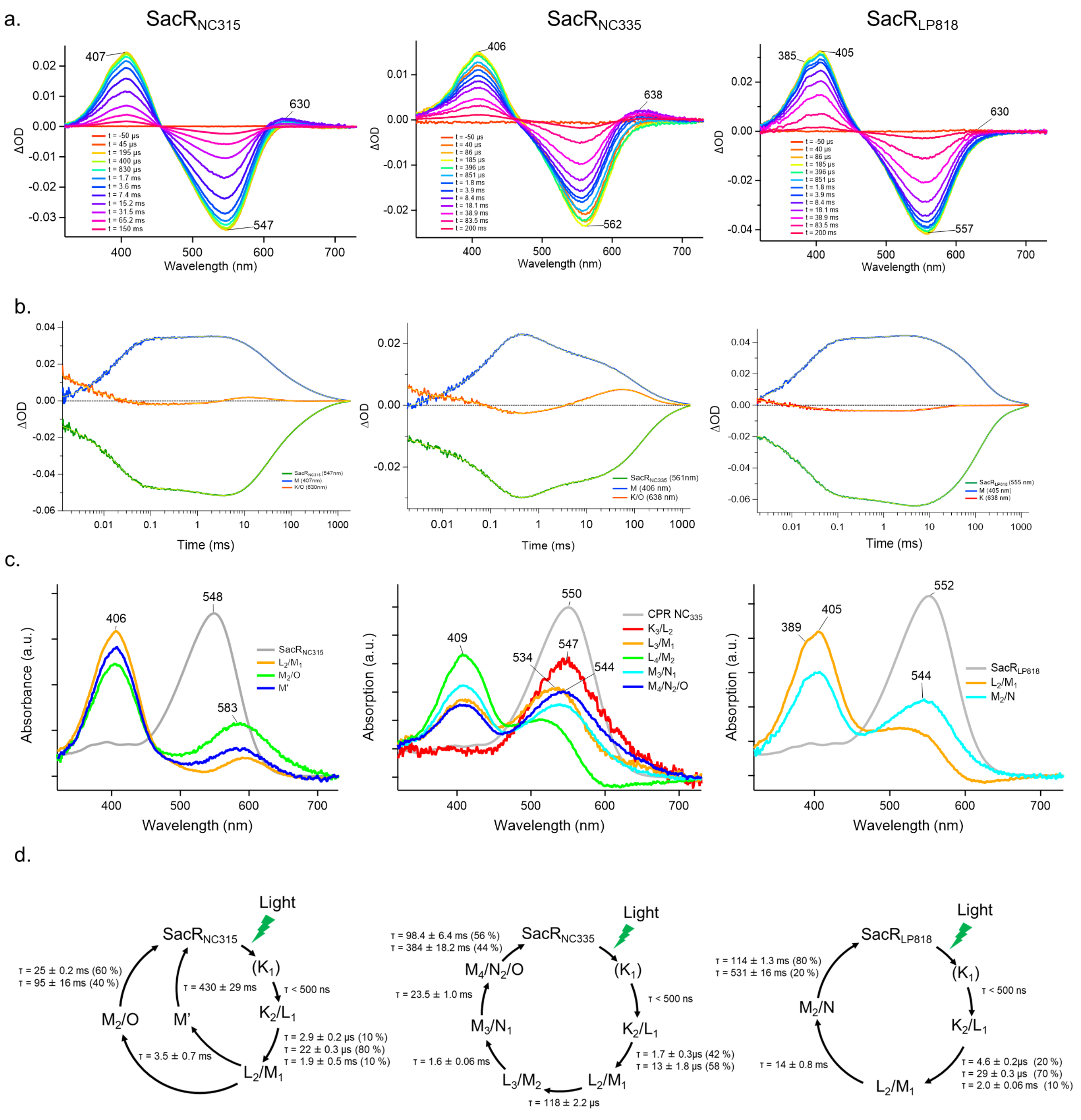

Figure S4. a) Transient absorption spectra of SacR$_{NC315}$ (left), SacR$_{NC335}$ (middle) and SacR$_{LP818}$ (right). **b**) Time evolution of transient absorption change of SacR$_{NC315}$ (left), SacR$_{NC335}$ (middle) and SacR$_{LP818}$ (right). The blue, green and orange lines represent the M intermediate, the breaching of the initial state, and the K or O intermediate, respectively. The yellow lines indicate fitting curves by multi-exponential function. **c)** Absorption spectra of photo-intermediates of SacR$_{NC315}$ (left), SacR$_{NC335}$ (middle) and SacR$_{LP818}$ (right). The absorption spectra of photo-intermediates were calculated from the decay-associated-spectra obtained by multi-exponential fitting according to previous study [24]. **d)** Kinetic models assuming sequential photocycles of SacR$_{NC315}$ (left), SacR$_{NC335}$ (middle) and SacR$_{LP818}$ (right) based on the fitting in **b**. The lifetime (τ) of each intermediate is indicated by numbers (mean ± S.D., the fraction of the intermediate decayed with each lifetime in its double exponential decay was indicated in parentheses).

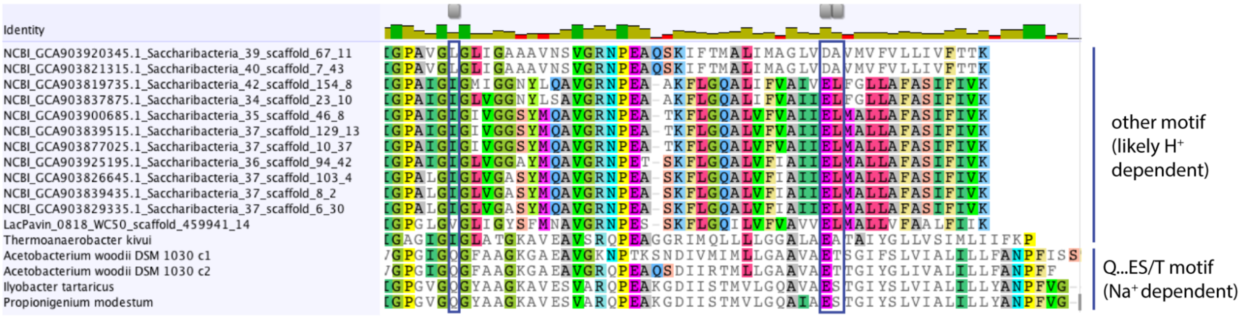

Figure S5. Partial protein alignment of the c subunit F_1F_o ATP synthase from Saccharibacteria with rhodopsin and known references. Columns encoding the ion binding motif are indicated by blue boxes.

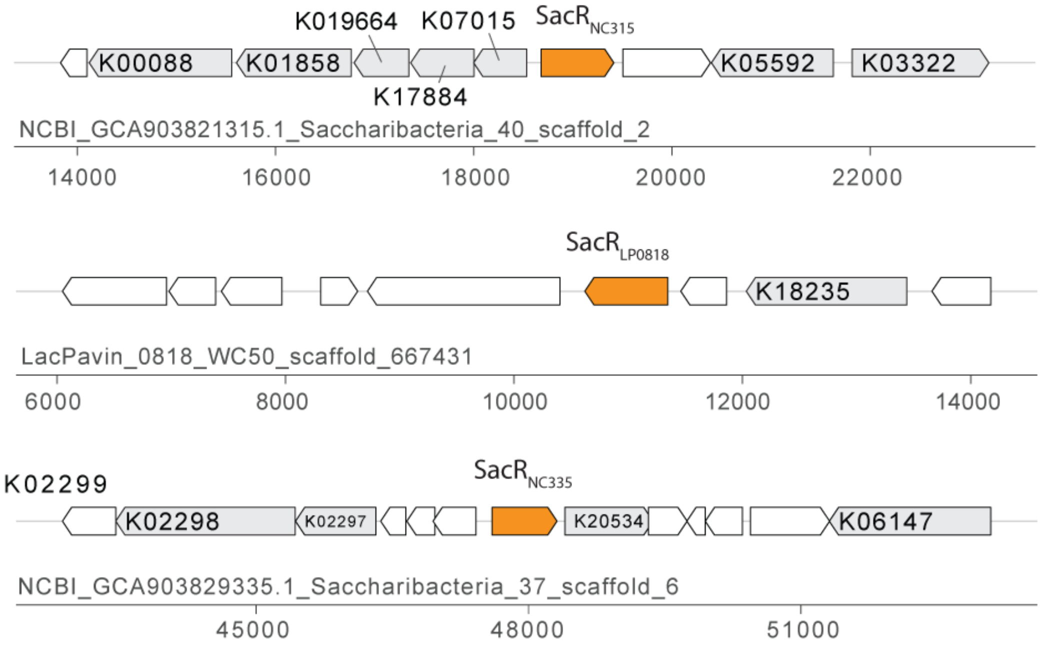

Figure S6. Immediate genomic context of the three characterized SacRs (orange). Neighboring genes with above-threshold KEGG annotations are indicated in gray with the highest-scoring HMM model. Genes without KEGG annotations are indicated in white.

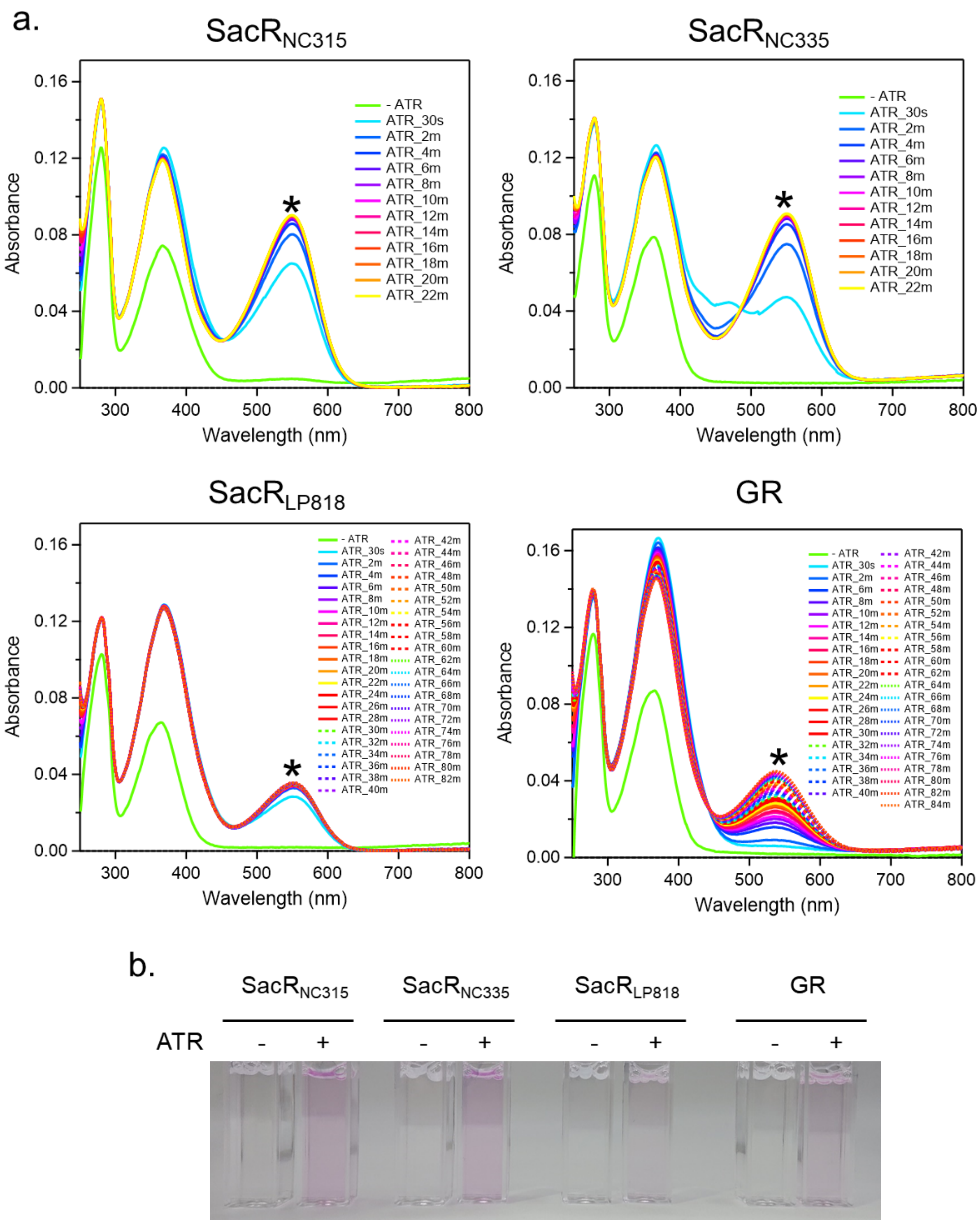

Figure S7. a) UV-visible absorption spectra showing the regeneration of retinal binding to SacRs and GR in 20 mM HEPES–NaOH, pH 7.0, 100 mM NaCl and 0.05% n-dodecyl-β-maltoside (DDM). The asterisks indicate absorption peaks of reconstituted rhodopsins. **b)** Appearance of SacRs and GR in solution without (−) and with (+) retinal (ATR). When supplemented with retinal, SacR and GR solutions were colored, indicating retinal regeneration.

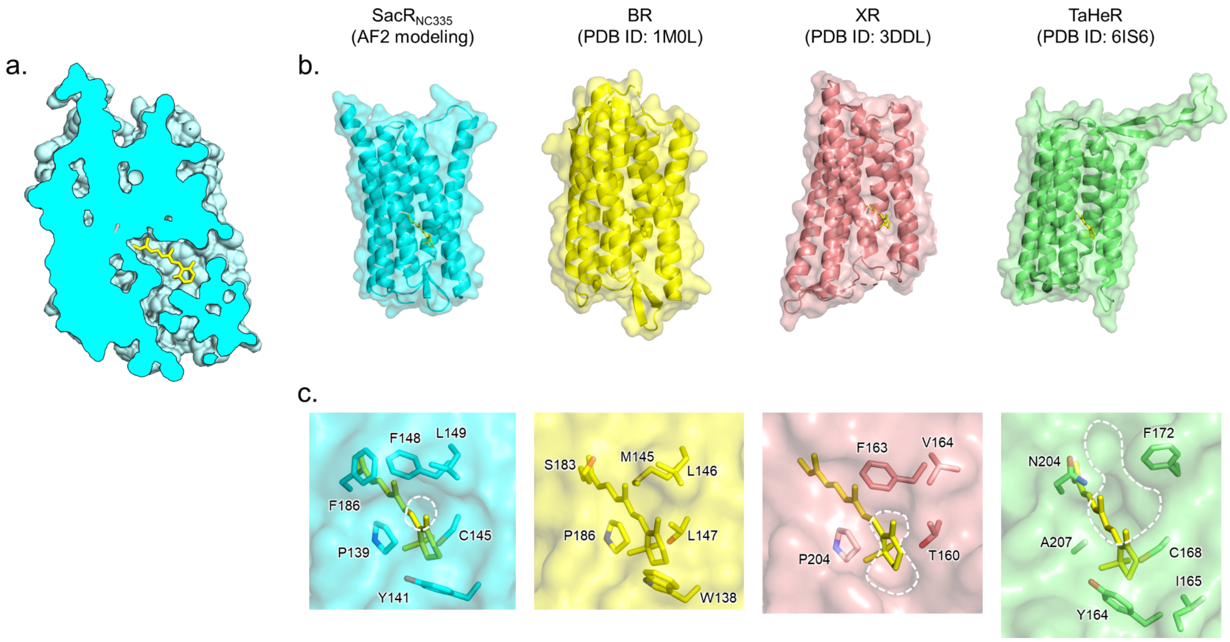

Figure S8. Modeled structure of SacR$_{NC335}$. **a)** cross-section view of a SacR$_{NC335}$ protomer modeled by Alphafold2 [15, 16]. **b**) Structural comparison of overall structures with other representative microbial rhodopsins. **c)** Magnified views of retinal-binding pockets with their molecular surfaces. The residues forming the β-ionone ring side of the retinal-binding pocket are indicated by stick models. White dashed circles represent the hydrophobic holes on the β-ionone ring side. No hydrophobic hole is present in the structure of BR.

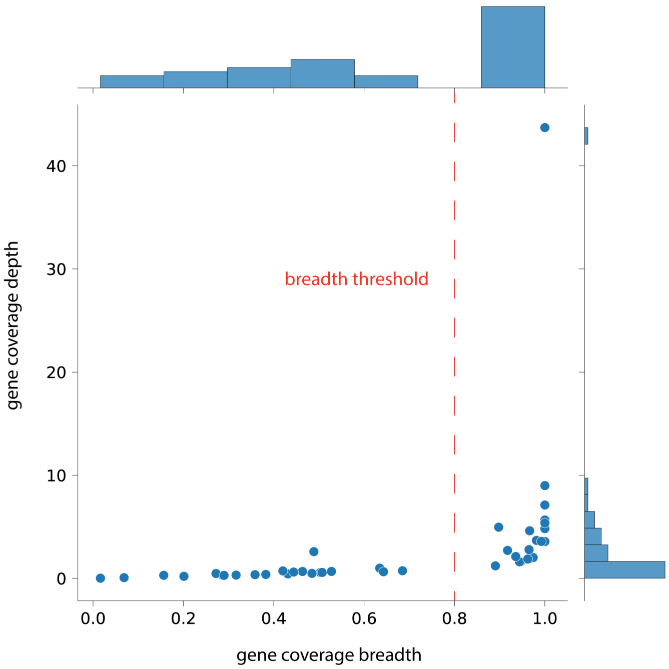

Figure S9. Sequencing coverage depth and coverage breadth (fraction of gene covered by reads) for SacRs in analyzed freshwater metagenomes. Metagenomic samples in which any SacR obtained 0.8 coverage breadth or greater were analyzed for genetic potential for retinal synthesis.

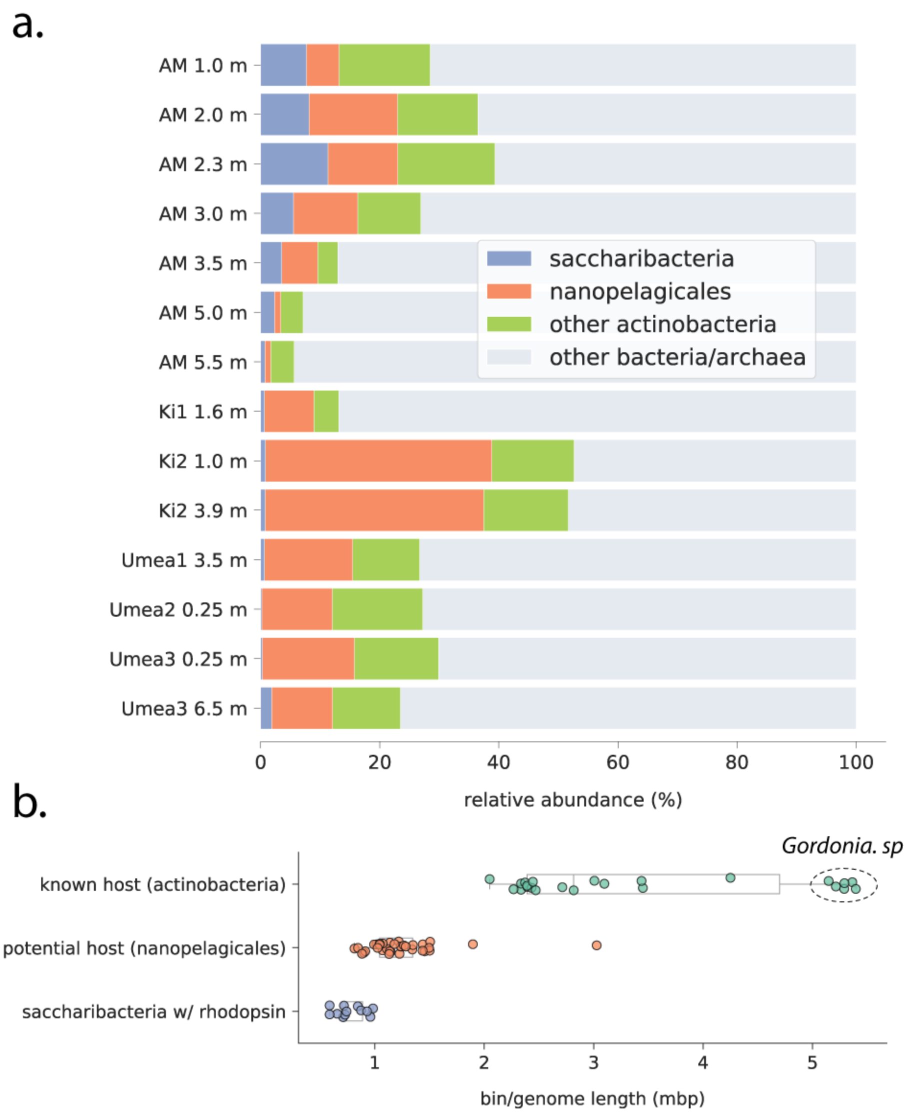

Figure S10. a) Relative abundance of Saccharibacteria, *Nanopelagicales* (potential Saccharibacteria hosts), and other Actinobacteria in the analyzed lake metagenomes. **b)** Genome size (for complete genomes) or bin length (for MAGs) for Saccharibacteria with rhodopsin, *Nanopelagicales* Actinobacteria (potential hosts), and Actinobacteria which are known to host Saccharibacteria in other environments.

References:

1. Jaffe AL, Thomas AD, He C, Keren R, Valentin-Alvarado LE, Munk P, et al. Patterns of Gene Content and Co-occurrence Constrain the Evolutionary Path toward Animal Association in Candidate Phyla Radiation Bacteria. *mBio* 2021; e0052121.
2. Chiriac M-C, Bulzu P-A, Andrei A-S, Okazaki Y, Nakano S, Haber M, et al. Ecogenomics sheds light on diverse lifestyle strategies in freshwater CPR. *Research Square*. 2021.
3. Edgar RC. Search and clustering orders of magnitude faster than BLAST. *Bioinformatics* 2010; **26**: 2460–2461.

4. Thompson JD, Gibson TJ, Higgins DG. Multiple sequence alignment using ClustalW and ClustalX. *Curr Protoc Bioinformatics* 2002; **Chapter 2**: Unit 2.3.
5. Minh BQ, Schmidt HA, Chernomor O, Schrempf D, Woodhams MD, von Haeseler A, et al. IQ-TREE 2: New Models and Efficient Methods for Phylogenetic Inference in the Genomic Era. *Mol Biol Evol* 2020; **37**: 1530–1534.
6. Langmead B, Salzberg SL. Fast gapped-read alignment with Bowtie 2. *Nat Methods* 2012; **9**: 357–359.
7. Konno M, Inoue K, Kandori H. Ion Transport Activity Assay for Microbial Rhodopsin Expressed in Cells. *Bio Protoc* 2021; **11**: e4115.
8. Hyatt D, Chen G-L, Locascio PF, Land ML, Larimer FW, Hauser LJ. Prodigal: prokaryotic gene recognition and translation initiation site identification. *BMC Bioinformatics* 2010; **11**: 119.
9. Aramaki T, Blanc-Mathieu R, Endo H, Ohkubo K, Kanehisa M, Goto S, et al. KofamKOALA: KEGG Ortholog assignment based on profile HMM and adaptive score threshold. *Bioinformatics* 2020; **36**: 2251–2252.
10. Hess V, Poehlein A, Weghoff MC, Daniel R, Müller V. A genome-guided analysis of energy conservation in the thermophilic, cytochrome-free acetogenic bacterium Thermoanaerobacter kivui. *BMC Genomics* 2014; **15**: 1139.
11. Buck M, Garcia SL, Fernandez L, Martin G, Martinez-Rodriguez GA, Saarenheimo J, et al. Comprehensive dataset of shotgun metagenomes from oxygen stratified freshwater lakes and ponds. *Sci Data* 2021; **8**: 131.
12. Olm MR, Crits-Christoph A, Bouma-Gregson K, Firek BA, Morowitz MJ, Banfield JF. inStrain profiles population microdiversity from metagenomic data and sensitively detects shared microbial strains. *Nat Biotechnol* 2021; **39**: 727–736.
13. Li D, Liu C-M, Luo R, Sadakane K, Lam T-W. MEGAHIT: an ultra-fast single-node solution for large and complex metagenomics assembly via succinct de Bruijn graph. *Bioinformatics* 2015; **31**: 1674–1676.
14. Buchfink B, Reuter K, Drost H-G. Sensitive protein alignments at tree-of-life scale using DIAMOND. *Nat Methods* 2021; **18**: 366–368.
15. Jumper J, Evans R, Pritzel A, Green T, Figurnov M, Ronneberger O, et al. Highly accurate protein structure prediction with AlphaFold. *Nature* 2021; **596**: 583–589.
16. Mirdita M, Schütze K, Moriwaki Y, Heo L, Ovchinnikov S, Steinegger M. ColabFold - Making protein folding accessible to all. *bioRxiv*.
17. Mirdita M, Steinegger M, Söding J. MMseqs2 desktop and local web server app for fast, interactive sequence searches. *Bioinformatics* 2019; **35**: 2856–2858.
18. UniProt Consortium. UniProt: a worldwide hub of protein knowledge. *Nucleic Acids Res* 2019; **47**: D506–D515.
19. Levy Karin E, Mirdita M, Söding J. MetaEuk-sensitive, high-throughput gene discovery, and annotation for large-scale eukaryotic metagenomics. *Microbiome* 2020; **8**: 48.
20. Mitchell AL, Almeida A, Beracochea M, Boland M, Burgin J, Cochrane G, et al. MGnify: the microbiome analysis resource in 2020. *Nucleic Acids Res* 2020; **48**: D570–D578.
21. Boyd JA, Woodcroft BJ, Tyson GW. GraftM: a tool for scalable, phylogenetically informed classification of genes within metagenomes. *Nucleic Acids Res* 2018; **46**: e59.
22. Crits-Christoph A, Diamond S, Al-Shayeb B, Valentin-Alvarado L, Banfield JF. A widely distributed genus of soil Acidobacteria genomically enriched in biosynthetic gene clusters. *bioRxiv*. 2021.
23. Batinovic S, Rose JJA, Ratcliffe J, Seviour RJ, Petrovski S. Cocultivation of an ultrasmall environmental parasitic bacterium with lytic ability against bacteria associated with wastewater foams. *Nat Microbiol* 2021; **6**: 703–711.
24. Chizhov I, Chernavskii DS, Engelhard M, Mueller KH, Zubov BV, Hess B. Spectrally silent transitions in the bacteriorhodopsin photocycle. *Biophys J* 1996; **71**: 2329–2345.
